# Dupuytren's Contracture: A Review of the Literature

**DOI:** 10.7759/cureus.74945

**Published:** 2024-12-02

**Authors:** Farihah Khaliq, Chijioke Orji

**Affiliations:** 1 Department of Trauma and Orthopedics, Aintree University Hospital, Liverpool, GBR; 2 Department of Orthopedics, Liverpool University Hospitals National Health Service (NHS) Foundation Trust, Liverpool, GBR

**Keywords:** dupuytren’s, dupuytren's contracture, dupuytren’s disease, dupuytren's fasciectomy, palmar fascia

## Abstract

Dupuytren's contracture is a chronic condition that affects the palmar fascia, leading to progressive flexion of the fingers, particularly the ring and little fingers. This article provides an in-depth review of the current understanding of the condition and its management. Commonly seen in older men of Northern European descent, Dupuytren's can significantly impair hand function as contractures develop. The underlying mechanisms involve complex cellular processes, particularly the role of transforming growth factor-beta in promoting fibroblast activity and collagen buildup. Treatment strategies depend on the severity of the condition; nonsurgical options like collagenase injections and needle aponeurotomy are effective for early stages but often have high recurrence rates. For more advanced cases, surgical options such as limited and radical fasciectomy are considered, aiming to restore finger extension while addressing potential complications. Challenges in management include high recurrence rates and variability in disease progression, emphasizing the need for standardized assessment protocols and innovative therapeutic approaches. This review underscores the importance of early diagnosis and intervention to preserve hand function and enhance quality of life. It calls for ongoing research into targeted therapies to reduce recurrence rates.

## Introduction and background

Dupuytren's contracture is a chronic, progressive fibroproliferative disorder affecting the palmar fascia, leading to permanent flexion deformities of the fingers. This condition was first identified by the French surgeon Baron Guillaume Dupuytren in 1831 [1]. It primarily affects the ring and little fingers, causing disability as the digits are progressively pulled into the palm. It is a relatively common condition, particularly among older men of Northern European descent, but its exact etiology remains to be understood [2]. While this disease is usually painless, the resultant contractures can severely impair hand function, reducing the ability to perform everyday tasks [1]. The underlying mechanisms of its development are complex and multifactorial, including genetic predisposition, environmental factors, and other associated comorbidities [3]. This review aims to provide a comprehensive overview of the current understanding of Dupuytren’s contracture.

## Review

Epidemiology

Dupuytren's contracture, often referred to informally as "Viking disease," is most commonly seen in people of Northern European descent. It has a particularly high prevalence in countries like Iceland, Norway, and the United Kingdom [4]. Research indicates that around 3%-6% of individuals of European ancestry are affected, with men being two to seven times more likely to develop the condition than women [5]. Typically, Dupuytren's contracture begins after the age of 40, and its incidence rises significantly with age; up to 20% of men over 60 may experience it. The condition tends to progress more aggressively in younger patients and those with a strong family history, leading to a higher chance of recurrence after treatment in these groups [5]. While Dupuytren's contracture is less frequent among individuals of African, Asian, or Hispanic descent, it can still occur in these populations [6]. The disease varies in severity, ranging from mild, asymptomatic nodules in the palm to more serious cases that result in debilitating contractures [2]. In advanced stages, the contracture can extend beyond the palm, making daily activities such as gripping things and writing very challenging [7].

Anatomy

Dupuytren's contracture primarily affects the palmar fascia, a dense connective tissue structure located beneath the skin of the palm. This fascia plays a critical role in supporting the tendons, nerves, and blood vessels of the hand. It is composed of several longitudinal and transverse fibers, with the pretendinous bands being the most significantly involved in Dupuytren's disease.

The palmar fascia is structured into three distinct layers: the superficial, intermediate, and deep layers. The superficial layer is anchored to the overlying skin and provides overall stability to the palm. This layer contains loose connective tissue, which allows for some mobility and flexibility while protecting deeper structures. The intermediate layer, or the palmar aponeurosis, features longitudinal fibers that extend distally into the fingers. These fibers are crucial for maintaining the structural integrity of the palm, allowing for both stability and functional movement [8].

The development of distinct deformities in Dupuytren's contracture is primarily driven by the pathological transformation of normal fascial bands into thickened, fibrotic cords. These cords progressively restrict normal hand movement. Central cords, which arise from the pretendinous bands, lead to the characteristic skin pitting and contracture of the metacarpophalangeal (MCP) joint. Natatory cords are typically responsible for the contracture of the web spaces between the fingers. However, the most clinically significant cords in the progression of the disease are the spiral cords, which can cause contractures at the proximal interphalangeal (PIP) joint.

Spiral cords originate from a combination of four key anatomical structures: the pretendinous band, the spiral band, the lateral digital sheath, and the Grayson ligament. Surgeons need to be particularly aware of how the spiral cord can lead to the displacement of the neurovascular bundle, which shifts centrally, superficially, and proximally within the affected digit [8,9].

Pathophysiology

The pathophysiology of Dupuytren's contracture is complex and involves several stages of cellular and biochemical changes in the palmar fascia. The disease progresses through three stages: the proliferative phase, the involutional phase, and the residual phase [10].

Proliferative Phase

This initial phase is characterized by an increase in the number of fibroblasts within the palmar fascia, resulting in the formation of nodules. These fibroblasts then differentiate into myofibroblasts, which are responsible for producing extracellular matrix components, particularly type III collagen. This process contributes to the creation of fibrous tissue. During this phase, there is significant collagen deposition and increased cellular activity within the affected fascia [11].

Involutional Phase

During this stage, myofibroblasts contract and align along the length of the fascia, leading to the thickening and shortening of the cords. As these cords become thicker and contract, they begin to pull the affected fingers into flexion, leading to the characteristic contractures seen in Dupuytren's disease.

Residual Phase

**Figure 1:**
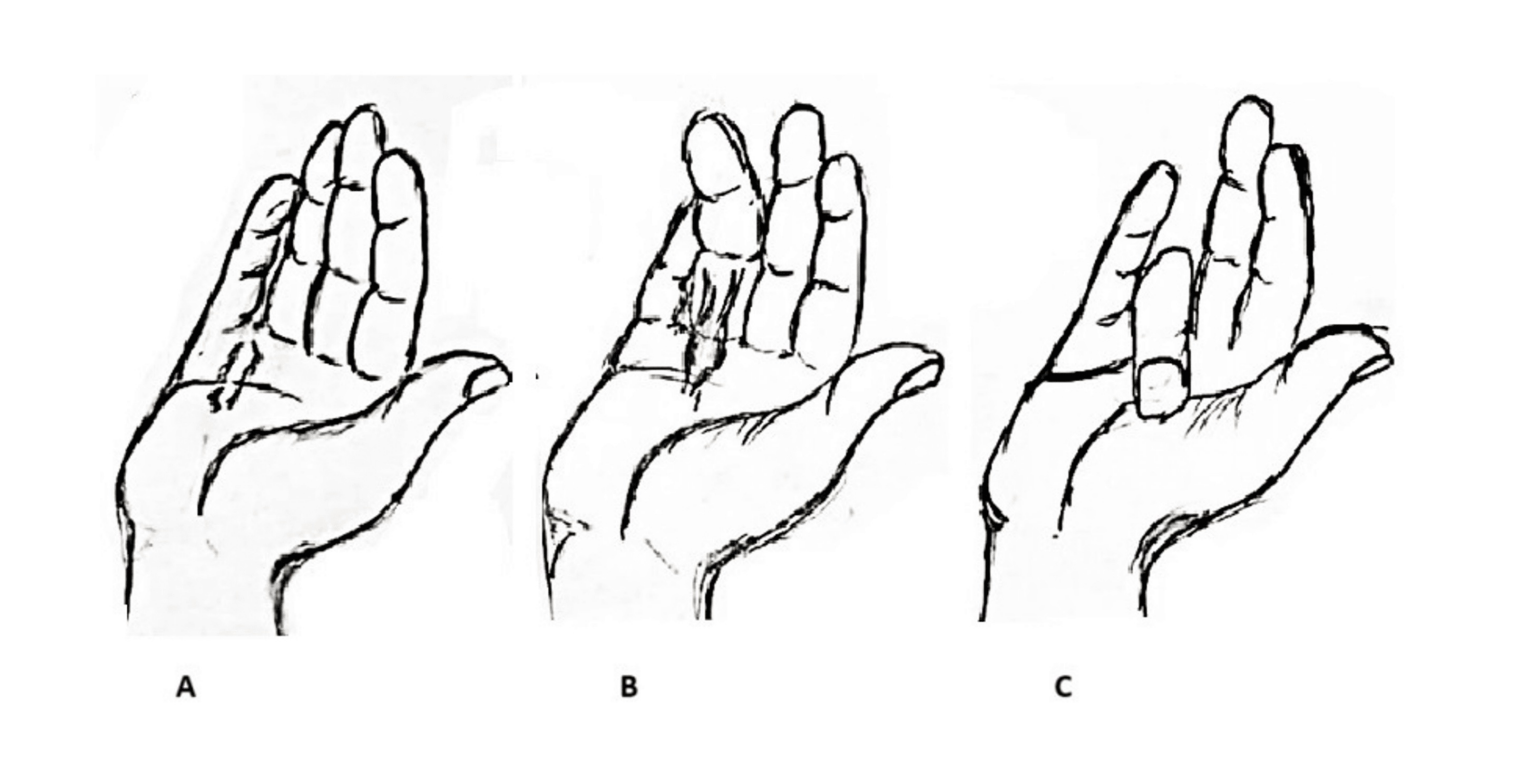
An illustration of the progression of Dupuytren's disease. Initially, there is skin dimpling due to the presence of a nodule (A). This progresses into a cord (B), eventually leading to contracture and flexion of the digit (C) Image credits: This is an original image created by the authors

In this final stage, the disease stabilizes, and while the cords become less cellular, they remain thickened and contracted. The flexion deformities become permanent at this point, and without intervention, the affected fingers stay curled into the palm [10]. Figure 1 illustrates the clinical progression of the disease in the hand.

Numerous molecular and biochemical pathways contribute to the development of Dupuytren's contracture. A key factor in this process is transforming growth factor-beta (TGF-β), a cytokine that stimulates fibroblast proliferation, myofibroblast differentiation, and collagen production. Elevated levels of TGF-β have been observed in the affected tissues of patients, indicating its significant role in driving the fibroproliferative response [12-14]. Additionally, other cytokines, such as platelet-derived growth factor and basic fibroblast growth factor, also play a role in the disease by promoting fibrosis and tissue remodeling [12].

Risk factors

Several important risk factors for Dupuytren's contracture have been identified. Genetic predisposition is significant, particularly among those of Northern European descent, and individuals with a first-degree relative affected by Dupuytren's disease have a significantly higher risk [3]. Advancing age is another well-known risk factor, with the majority of cases occurring after the age of 40 and prevalence increasing significantly in older populations. Furthermore, men are disproportionately affected; studies suggest that they are two to seven times more likely to develop the condition than women and that it tends to progress more rapidly and aggressively in men [15,16]. Lifestyle factors also play an important role, such as smoking and alcohol consumption, both of which have been correlated with an increased risk. Comorbidities like diabetes and epilepsy, specifically those on anticonvulsant therapy, also contribute to the likelihood of developing Dupuytren's contracture [17].

Differential diagnosis

The clinical presentation of Dupuytren's contracture can sometimes be mistaken for other conditions that lead to contractures or deformities of the hand and fingers. Key differential diagnoses to consider include trigger finger, also known as stenosing tenosynovitis, which involves inflammation and narrowing of the tendon sheath, causing the affected finger to lock or catch in a flexed position. This condition primarily affects the flexor tendons and presents with intermittent locking, while Dupuytren's contracture specifically involves the palmar fascia, resulting in a fixed contracture. Another possibility is flexor tendon contracture, which can arise from trauma or injury to the flexor tendons, leading to scarring and subsequent contracture. Unlike Dupuytren's, which targets the fascia, flexor tendon contractures mainly involve the tendons themselves. Ulnar nerve entrapment, or cubital tunnel syndrome, may also be considered, as it can cause clawing of the ring and little fingers due to compression of the ulnar nerve at the elbow, potentially resembling Dupuytren's contracture [18]. However, this condition typically presents with sensory changes, such as numbness and tingling, alongside weakness in the hand, which are not seen in Dupuytren's. Plantar fibromatosis, or Ledderhose disease, is similar to Dupuytren's but affects the plantar fascia of the feet. In some cases, patients may experience both plantar and palmar fibromatosis, making it crucial to differentiate between the two for appropriate treatment [19].

Assessment tools and grading systems

A variety of assessment tools and grading systems have been developed to evaluate disease severity and progression, each offering distinct advantages in both clinical practice and research settings. The Tubiana Classification is one of the most widely utilized grading systems for Dupuytren's disease. It categorizes the disease based on the degree of flexion contracture at the MCP and PIP joints. Patients are classified into four stages, from stage 0 (no contracture) to stage IV (severe contracture with joint flexion greater than 135°). Table 1 outlines Tubiana's original classification, which has been modified by others. This classification is particularly useful in assessing the overall severity of the disease and informing treatment decisions. However, it may be less effective in the early stages of the disease, where contractures are minimal or not yet present [20].

**Table 1:** Tubiana's classification system of Dupuytren's contracture. The total extension deficit is calculated by combining the deficit at the MCP, PIP, and DIP joints MCP: metacarpophalangeal; PIP: proximal interphalangeal; DIP: distal interphalangeal Source: [21]

Stage	Extension deficit
0	No extension deficit
N	Nodule without contracture
I	Total extension deficit (MCP + PIP + DIP) between 0° and 45°
II	Total extension deficit (MCP + PIP + DIP) between 45° and 90°
III	Total extension deficit (MCP + PIP + DIP) between 90° and 135°
IV	Total extension deficit (MCP + PIP + DIP) > 135°

The Disabilities of the Arm, Shoulder, and Hand (DASH) Questionnaire is a widely used patient-reported outcome measure (PROM) that assesses functional impairment and the impact of hand dysfunction on daily activities. While it is not disease-specific, the DASH provides valuable insight into the broader functional limitations caused by Dupuytren's disease. It is commonly employed in both pretreatment and posttreatment evaluations, offering a comprehensive view of the patient's perceived outcomes beyond clinical signs.

Range of motion (ROM) measurements are another cornerstone in assessing Dupuytren's disease, particularly with regard to the degree of joint flexion and extension. ROM is typically measured at the MCP and PIP joints using a goniometer for accuracy. These measurements provide an objective, quantitative assessment of contracture severity and are essential for monitoring disease progression and evaluating the effectiveness of treatment over time.

In recent years, emerging imaging techniques, such as ultrasound and magnetic resonance imaging (MRI), have been explored as tools for assessing the underlying pathology of Dupuytren's disease. Ultrasound imaging is particularly useful in identifying nodules and cords within the palmar fascia, offering a detailed view of the fibrotic change's characteristic of the disease. MRI, while less commonly used in routine clinical practice due to its cost and limited accessibility, provides high-resolution images of soft tissues and may help in early diagnosis and treatment planning, especially in complex or advanced cases.

Additionally, PROMs such as the Michigan Hand Outcomes Questionnaire and the Patient Evaluation Measure are increasingly used to assess patient satisfaction, functional status, and overall quality of life. These instruments help capture the psychosocial impact of Dupuytren's disease and are often incorporated into clinical trials and longitudinal studies to evaluate treatment efficacy from the patient's perspective [20,22].

Nonsurgical management

The management of Dupuytren's contracture is influenced by the stage and severity of the disease, as well as the patient's functional limitations and personal preferences. In the early stages, when contractures are mild and hand function is largely intact, nonsurgical interventions are usually considered. These treatments aim to slow disease progression and enhance functionality but do not provide a cure.

One option is collagenase *Clostridium histolyticum* (CCH), a minimally invasive treatment involving the injection of an enzyme derived from the bacterium *C. histolyticum*. This enzyme specifically targets and breaks down collagen in the fibrous cords, effectively weakening the contracture. Approved by the FDA in 2010, CCH has shown efficacy in clinical trials, reducing contracture and improving hand function [23]. After the injection, a manipulation procedure is performed to rupture the weakened cord. Success rates for CCH range from 65% to 85%, though some studies report recurrence rates of 35%-40% within five years [24,25]. Side effects may include pain, swelling, bruising, and a risk of tendon rupture; nevertheless, CCH remains an effective nonsurgical option for patients with moderate contractures.

Another minimally invasive technique is needle aponeurotomy or percutaneous needle fasciotomy. This procedure uses a small needle to sever the fibrous cords beneath the skin, allowing the affected finger to straighten. Typically performed under local anesthesia in an outpatient setting, needle aponeurotomy offers a shorter recovery time compared to surgical interventions. It is especially effective for patients in the early stages of Dupuytren's contracture and has high rates of patient satisfaction [26]. However, like CCH, it has a relatively high recurrence rate, with up to 85% of patients experiencing a return of contracture within five years. Despite this, it remains a viable option for those seeking a less invasive treatment.

Corticosteroid injections are another option used in the early stages of Dupuytren's disease to reduce inflammation and slow contracture progression. This treatment is based on the understanding that inflammation contributes to the early stages of fibrosis, and corticosteroids can inhibit fibroblast proliferation and collagen deposition. While some studies suggest that steroid injections can reduce nodule size and alleviate pain, they do not prevent contracture formation. Additionally, the effects of these injections are typically temporary, necessitating repeated treatments [27].

Radiotherapy has also been explored as a treatment for early-stage Dupuytren's contracture, particularly in Europe. Low-dose radiotherapy targets proliferating fibroblasts in the palmar fascia and can help halt disease progression. A 2016 study indicated that radiotherapy could prevent disease advancement in about 70% of patients, with minimal side effects. However, this treatment is generally reserved for cases with significant concern about disease progression, and its long-term efficacy is still being studied [28].

Physical therapy and splinting are often used as adjunctive treatments for Dupuytren's contracture. While these interventions may provide temporary symptom relief and improve ROM, their long-term effectiveness in preventing disease progression or recurrence is limited. In some instances, splinting may be employed postsurgically to maintain finger extension, but prolonged splinting is generally discouraged due to the risk of increasing stiffness.

Surgical management

Surgical intervention is regarded as the primary treatment option for patients with advanced Dupuytren's contracture, especially those experiencing significant contractures that hinder hand function. The main objectives of surgery are to release the contracted fascia, restore finger extension, and enhance overall hand function. The choice of surgical procedure depends on the severity of the condition, the presence of comorbidities, and the patient's overall health.

Fasciectomy is the most commonly performed surgical procedure for Dupuytren's contracture, involving the removal of the affected fascia. There are two main types of fasciectomy: limited fasciectomy and radical fasciectomy [1,5].

Limited fasciectomy involves excising only the affected cords and nodules while preserving surrounding healthy tissue. This less invasive approach typically results in a shorter recovery time and is associated with good functional outcomes and lower complication rates, making it the preferred surgical option [1]. However, recurrence remains a concern, with studies indicating that up to 50% of patients may experience a return of contracture within five to ten years [29].

Radical fasciectomy, on the other hand, entails the complete removal of the palmar fascia, including both affected and unaffected tissue. This more extensive procedure is usually reserved for severe, recurrent cases of Dupuytren's contracture. While it tends to have lower recurrence rates compared to limited fasciectomy, radical fasciectomy carries a higher risk of complications such as nerve damage, hematoma formation, and delayed wound healing [19]. Therefore, it is typically considered only when less invasive options are unlikely to succeed.

Dermofasciectomy is another surgical option that involves removing both the affected fascia and the overlying skin. Following the excision, a skin graft is used to cover the resulting defect. This procedure is generally reserved for cases with significant skin involvement or where there is a heightened concern about recurrence, as it has been shown to lower the likelihood of contracture recurrence [29]. However, dermofasciectomy is more complex than standard fasciectomy, resulting in a longer recovery time and a greater risk of complications, including graft failure and infection.

Enzyme fasciotomy is a minimally invasive technique that involves injecting collagenase into the fibrous cords to break down collagen, facilitating the manual manipulation of the finger to straighten it. Although this procedure is often categorized as nonsurgical management, it requires a level of expertise and patient selection typically associated with surgical interventions. It is effective in moderate cases, but recurrence rates remain challenging, and enzyme fasciotomy is less effective in patients with severe contractures affecting multiple digits [30].

The absence of a clear and standardized definition of recurrence represents a significant limitation in the reported rates of Dupuytren's disease recurrence across the literature. In the absence of a consensus on what constitutes recurrence, studies often rely on varying criteria, resulting in inconsistent and highly variable recurrence rates. This subjectivity in defining recurrence contributes to a broad range of reported rates, from 0% to 85%, depending on the specific criteria used, the patient population, and the type of surgery performed. Establishing a unified definition is critical to producing more reliable, comparable data on recurrence rates, thereby ensuring that findings across studies are meaningful and can inform clinical practice. For example, Felici et al. defined recurrence as a passive extension deficit greater than 20° in at least one treated joint, accompanied by a palpable cord, when compared to measurements taken 6-12 weeks after surgery. In contrast, other studies may use alternative criteria, such as changes in the degree of contracture, reappearance of nodules, or the presence of symptoms like pain or functional limitation [31].

Challenges in the management and assessment of Dupuytren's disease

Managing Dupuytren's disease involves a complex set of challenges that can hinder treatment outcomes and affect patient satisfaction. A key issue is the notably high recurrence rate associated with both nonsurgical and surgical treatments. Studies show that while nonsurgical options like collagenase injections and needle aponeurotomy can achieve favorable short-term results, recurrence rates on average are around 35%-58% within five years [31,32]. This pattern emphasizes the need for long-term monitoring and repeat interventions, which can strain healthcare resources and lead to patient frustration, complicating the overall management strategy for clinicians.

Another challenge lies in the variability of disease progression and treatment responses among patients. Factors such as genetic predisposition, age, and comorbid conditions like diabetes significantly influence this variability. For example, individuals with a family history of Dupuytren's disease often experience more aggressive progression, necessitating a tailored treatment approach [3]. Clinicians face the challenge of weighing the risks and benefits of different treatment options, carefully considering each patient's unique circumstances when making decisions.

Additionally, the pathophysiology of Dupuytren's disease, which involves dysregulated fibroblast activity and abnormal collagen deposition, highlights the need for innovative therapies targeting these underlying molecular processes. Current treatments often fall short in addressing these issues, revealing a critical gap in therapeutic effectiveness and a clear demand for research into targeted biological therapies.

Patient adherence to treatment protocols and follow-up care also presents a significant hurdle. Many patients may not fully grasp the progressive nature of Dupuytren's disease, leading to an underestimation of the need for consistent monitoring and proactive interventions. This lack of understanding can create care gaps, resulting in worsening contractures that could have been prevented with timely action [22,25].

There is an urgent need for standardized assessment tools and grading systems that accurately capture the severity and progression of Dupuytren's disease, enhancing treatment planning and outcome evaluation. The absence of universally accepted criteria complicates comparisons of clinical outcomes across studies and weakens the evidence base guiding clinical practice [33].

While several standardized assessment tools have been developed to evaluate Dupuytren's disease, current literature highlights the need for further validation and standardization to ensure these instruments are applied consistently across clinical settings. One key concern is the variability in their application, which can undermine the reliability of results. For example, tools like the total passive extension test, which is commonly used to measure the degree of contracture, may be performed using different techniques or measurement protocols. This lack of uniformity can lead to discrepancies in reported severity, making it challenging to compare outcomes across different clinics or studies [34,35].

Furthermore, many of these assessment tools have not been comprehensively validated across diverse patient populations. This raises questions about their generalizability and their ability to capture the full spectrum of disease progression in various ethnic and demographic groups. Without standardized protocols, it becomes difficult to make meaningful comparisons of treatment efficacy or recurrence rates. Moreover, while certain tools are effective at quantifying contracture severity, their ability to correlate with functional outcomes and quality of life remains unclear. This gap in understanding underscores the need for a more holistic approach that links clinical measures with patient-reported outcomes, particularly given the significant impact Dupuytren's disease can have on daily function and psychosocial well-being [34,36].

The variability in how clinicians interpret assessment results is another critical challenge. Differences in individual experience and training can lead to discrepancies in how severity is judged, contributing to reduced interrater reliability. Improving the consistency of clinician assessments would help reduce these inconsistencies, ensuring that patients receive the most appropriate treatment. Enhanced standardization of assessment tools could ultimately enable more personalized and precise treatment plans, allowing clinicians to better determine when nonsurgical options are appropriate versus when surgery may be necessary. It would also improve the monitoring of disease progression over time, providing a clearer picture of treatment effectiveness.

## Conclusions

Dupuytren's contracture is a complex and progressive condition that significantly impacts hand function, particularly among individuals of Northern European descent. Understanding its anatomy, epidemiology, and underlying pathophysiology is crucial for effective diagnosis and treatment. While nonsurgical options like collagenase injections and needle aponeurotomy offer promising results for early-stage disease, the high recurrence rates highlight the need for ongoing management and monitoring. Surgical interventions remain the primary approach for advanced cases, aiming to restore function and improve quality of life.

As research progresses, there is a clear imperative for the development of targeted therapies that address the molecular mechanisms driving the disease. Furthermore, enhancing standardized assessment tools will facilitate more consistent evaluations of severity and treatment outcomes. By fostering a comprehensive understanding of Dupuytren's contracture and its management, we can better support affected individuals and improve their overall well-being. Continued exploration in this field is essential to advance therapeutic options and ultimately reduce the burden of this condition.
